# Familial glucocorticoid deficiency due to a novel *TXNRD2* variant: expanding the spectrum of a rare genetic cause

**DOI:** 10.3389/fendo.2026.1834727

**Published:** 2026-05-28

**Authors:** Ibrahim Al Alwan, Kheloud M. Alhamoudi, Abdullah Ibrahim Alzaben, Beshaier Almulhem, Nawal Qawasmi, Meshael Alswailem, Sara Alotaibi, Burair Alsaihati, Amjad Jabaan, Moeber Mahzari, Christa E. Flück, Ali S. Alzahrani

**Affiliations:** 1 College of Medicine, King Saud Bin Abdulaziz University for Health Sciences, Riyadh, Saudi Arabia; 2 Department of Pediatrics, King Abdullah International Medical Research Center (KAIMRC), Riyadh, Saudi Arabia; 3 Pediatric Endocrinology Division, Department of Pediatrics, King Abdullah Specialist Children’s Hospital (KASCH), King Abdulaziz Medical City, Ministry of National Guard Health Affairs (MNG-HA), Riyadh, Saudi Arabia; 4 Department of Molecular Oncology, King Faisal Specialist Hospital & Research Centre, Riyadh, Saudi Arabia; 5 College of Medicine, Alfaisal University, Riyadh, Saudi Arabia; 6 Department of Pediatrics, Imam Abdulrahman Bin Faisal University, College of Medicine, Dammam, Saudi Arabia; 7 Applied Genomic Technologies Institute, King Abdulaziz City for Science and Technology, Riyadh, Saudi Arabia; 8 Department of Medicine, Ministry of the National Guard-Health Affairs, Riyadh, Saudi Arabia; 9 Department for Biomedical Research, University Hospital Bern, Bern, Switzerland; 10 Department of Medicine, King Faisal Specialist Hospital & Research Centre, Riyadh, Saudi Arabia

**Keywords:** Adrenal insufficiency, familial glucocorticoid deficiency, missense mutation, *TXNRD2*, whole genome sequencing

## Abstract

**Background:**

Familial glucocorticoid deficiency (FGD) is a rare autosomal recessive disorder characterized by isolated cortisol deficiency and elevated adrenocorticotropic hormone (ACTH) levels. Variants in *TXNRD2*, which encodes mitochondrial thioredoxin reductase 2, have recently been implicated in FGD; however, the phenotypic and mutational spectrum remain extremely limited.

**Methods:**

Whole-genome sequencing (WGS) was performed in a proband from a Saudi family who presented in early childhood with clinical and biochemical features consistent with FGD (Low basal and stimulated cortisol of < 5 nmol/l and extremely elevated ACTH levels of > 2500 pg/ml) and seizure disorder requiring medical treatment and with basal ganglia changes noted on brain MRI at presentation. WGS identified a novel likely pathogenic *TXNRD2* variant with no other potential variant in FGD-associated genes. Population frequency, segregation analysis, evolutionary conservation, and *in silico* pathogenicity predictions were assessed for the identified variant.

**Results:**

WGS identified a novel missense homozygous *TXNRD2* (NM_001282512) variant (c.575C>T (p.Pro192Leu) in the proband, and in the heterozygous state in both parents, supporting autosomal recessive inheritance. This variant has not been reported in a local population database of > 18000 exomes, is extremely rare in international population databases (minor allele frequency 0.00000479), and affects a highly conserved residue. The variant was consistently predicted by multiple *in silico* tools to have deleterious effects on protein structure and function. No pathogenic, likely pathogenic, or VUS was found in other genes involved in FGD, including *MC2R, MRAP, STAR, CYP11A1, NNT, MCM4, or SGPL1.*

**Conclusion:**

This report adds another patient with a novel variant to the few previously described patients and expands the genetic spectrum of the very rare *TXNRD2*-associated FGD, supporting the role of mitochondrial redox dysregulation in adrenal insufficiency.

## Introduction

Familial glucocorticoid deficiency (FGD) is a group of rare autosomal recessive inherited disorders of primary adrenal insufficiency (1). The hallmark of these disorders is adrenocortical deficiency, mostly involving the glucocorticoid but sometimes the mineralocorticoid axis as well (2).

Over the last few decades, significant advances in molecular genetics have transformed the understanding of FGD from a clinically defined entity into a genetically heterogeneous disorder (3). Many genes and genetic variants affecting ACTH signaling, adrenal steroidogenesis, mitochondrial redox balance, and cellular metabolism have been identified (3–5). These discoveries have revealed that FGD is not simply a disorder of ACTH resistance, but a more complex group of disorders affecting adrenocortical cells in various ways, including mitochondrial dysfunction, oxidative stress, and impaired cholesterol transport (3, 4, 6). To date, mutations in the ACTH receptor gene (*MC2R*) or its accessory protein (*MRAP*), account for approximately 40–50% of reported cases (6, 7). Additional rarely mutated genes including cellular redox status genes such as nicotinamide nucleotide transhydrogenase (*NNT)*, Thioredoxin reductase 2 (*TXNRD2)*, steroidogenic acute regulatory protein (*StAR)*, cytochrome P450 side-chain cleavage enzyme (*CYP11A1)*, sphingolipid deficiency gene (*SGPL1)*, and defective DNA replication gene (*MCM4)* have expanded the phenotypic and mechanistic spectrum of the disease (3). Excluding *MC2R* and *MRAP*, genetic alterations in these genes have rarely been reported.

*TXNRD2* encodes mitochondrial thioredoxin reductase 2, a key enzyme that maintains mitochondrial redox balance and protects steroidogenic tissues from oxidative stress. It is part of the thioredoxin system, which consists of thioredoxin (TXN), thioredoxin reductase (TXNRD), and NADPH. Within mitochondria, TXNRD2 reduces oxidized thioredoxin-2, thereby protecting cells from oxidative stress and regulating redox-dependent signaling pathways. Given the high oxidative burden associated with adrenal steroidogenesis, impairment of TXNRD2 function provides a plausible pathogenic mechanism for adrenal insufficiency. Germline *TXNRD2* variants are rare causes of FGD and have been primarily reported, mostly in heterozygous forms in patients with primary open-angle glaucoma or dilated cardiomyopathy (8–11). Only four biallelic FGD-associated *TXNRD2* variants have been reported so far in a total of 12 patients, underscoring the rarity of this genetic subtype (10, 12–15) (**Table 1**). In this report, we expand the molecular and mechanistic spectrum of *TXNRD2*-related familial glucocorticoid insufficiency through identification of a novel *TXNRD2* variant affecting mitochondrial redox homeostasis.

**Table 1:** Previously described cases and the current case with familial glucocorticoid deficiency due to *TXNRD2* mutations.

Mutation	Protein change	Sex	Exon	Zygosity	Ethnicity	Reference
c.1341T>G	p.Y447X	F	15	homozygous nonsense variant	Kashmiri	(13)
c.1341T>G	p.Y447X	F	15	homozygous nonsense variant	Kashmiri	(13)
c.1341T>G	p.Y447X	F	15	homozygous nonsense variant	Kashmiri	(13)
c.1341T>G	p.Y447X	F	15	homozygous nonsense variant	Kashmiri	(13)
c.1341T>G	p.Y447X	F	15	homozygous nonsense variant	Kashmiri	(13)
c.1341T>G	p.Y447X	F	15	homozygous nonsense variant	Kashmiri	(13)
c.1341T>G	p.Y447X	F	15	homozygous nonsense variant	Kashmiri	(13)
c.1341T>G	p.Y447X	F	15	Unclear (Not mentioned)	South India	(14)
c.1081G>A	p.V361M	M	12	Homozygous missense variant	Pakistani	(10)
c.1348-1G>T	p.M450Vfs*20	M	15	homozygous splice variant	Moroccan	(12)
c.1391A > G c.1141C > T	p.H464R p.R381W	M	16 13	compound heterozygous variants	Chinese	(15)
c.C575T	p.P192L	M	7	Homozygous missense	Saudi	This Report

## Materials and methods

### The patient

The patient is a 28-year-old Saudi male, who was born to consanguineous parents. He was apparently healthy until the age of 3 years and 3 months, when he was hospitalized for a generalized afebrile tonic-clonic seizure following an upper respiratory infection. Although the initial clinical assessment revealed only severe dehydration, he suffered a second convulsion and a decreased level of consciousness that necessitated intubation and mechanical ventilation for one week.

Initial laboratory investigations were significant only for a low serum sodium of 124 mmol/L (135-145), with normal serum potassium of 4.6 nmol/l (3.5-5.0) and normal blood counts, serum chemistry and plasma glucose level. Blood and cerebrospinal fluid cultures were negative. Following a successful extubation, the patient exhibited persistent hyponatremia and a single episode of hyperkalemia 5.6 mmol/l (3.4-4.7), raising suspicion of primary adrenal insufficiency. This diagnosis was confirmed by a morning cortisol level of less than 5 nmol/L (normal range, 119-610), a markedly elevated ACTH level exceeding 2500 pg/mL (normal range 10–46 pg/mL), and an ACTH stimulation test, which showed a cortisol post-stimulation levels of less than 5 nmol/L with a normal 17-hydroxyprogesterone levels. At age 4 years, renin and aldosterone levels were measured and were found normal for age.

Further diagnostic workup included a normal adrenal ultrasound with normal size of the adrenal glands, absence of hemorrhage and calcifications and negative results for adrenal antibodies. CT scan of the adrenal glands was not done to avoid radiation and due to the high diagnostic utility of adrenal ultrasonography in children (16). Very long-chain fatty acids level was normal. A brain MRI at the age of 4 years showed abnormalities in the basal ganglia (swollen and significantly hyperintense on T2-weighted images) of unclear cause. A repeated brain MRI at the age of 25 years did not show this finding.

The patient was stabilized on hydrocortisone 12 mg/m2/day and fludrocortisone 0.1 mg daily for adrenal replacement and carbamazepine for seizure management. Fludrocortisone was stopped later on and the patient remained stable on hydrocortisone. Over a 17-year follow-up period, he remained seizure-free for the past 9 years and demonstrated normal pubertal development. Despite receiving adequate doses of hydrocortisone, he maintained persistent hyperpigmentation and persistently high ACTH levels ranging from 534 pg/ml to > 5000 pg/ml. His height and weight were at the 10th and 70th percentiles, respectively. His motor, visual, and auditory functions are normal, but his cognitive function as an adult is clearly subnormal and has significantly affected his academic performance as he only completed primary school. He lives with his parents and is not married, additionally, he is not able to retain employment. His current thyroid, liver, renal, and cardiac functions are normal, including a normal echocardiogram.

Family history is unremarkable apart from first-degree consanguinity of parents, with no similar conditions reported in the patient’s siblings or extended family. There is no family history of unexpected deaths at a young age and no history of early cardiac disease or glaucoma.

His current height is 167 cm, weight 93kg, and BMI 33.3 kg/m2. He has generalized hyperpigmentation involving the skin and mucous membranes. His latest laboratory investigations revealed normal Na+, K+, Co2, Creatinine, Testosterone, LH and FSH. ACTH was elevated at >2000 pg/ml. His current hydrocortisone dose is 12 mg/m^2^/day.

### Genomic DNA extraction

Genomic DNA (gDNA) was extracted from the peripheral blood cells of the patient and parents using a commercial DNA extraction kit (QIAamp Blood Midi Kit, Qiagen, Hilden, Germany) according to the manufacturer’s instructions. The extracted DNA was then quantified using a Nanodrop‐1000 spectrophotometer.

### Next-generation sequencing and variant interpretation

The target sequences of the gDNA sample was fragmented by sonication, and Illumina adapters were ligated to the generated fragments for subsequent sequencing on the HiSeqX platform (Illumina Inc., San Diego, CA, USA) to yield an average coverage depth of ~30X. Sequence data were mapped to the human genome build UCSC hg19. We used the same protocol for whole genome sequencing (WGS) as previously described (17). Data were analyzed using an in-house variant interpretation pipeline. The in-house databases include collections of known disease-causing variants in the KSA population, maintained by the Center for Genomic Medicine (CGM-DB). All disease-causing variants reported in the Human Gene Mutation Database (HGMD) (18) and ClinVar (19), in addition to all variants with minor allele frequency below 1% in the genomAD (http://gnomad.broadinstitute.org) were given priority. We also focused on genes that are known to cause FGD and adrenal insufficiency, including *MC2R, MRAP, STAR, CYP11A1, NNT, TXNRD2, MCM4, SGPL1, CDKN1C, AAAS, DAX1, NR5A1, ABCD1, VPS13B, VPS13C, NR0B1*, and *NR3C1.*

### Database screening and *in-silico* analysis

To predict the pathogenicity and the potential effects of the candidate variant, *in-silico* computational prediction tools including Provean (https://www.jcvi.org/research/provean) (20), SIFT (https://siftdna.org/) (21), MutationTaster (https://www.mutationtaster.org/) (22), and PolyPhen2 (available at https://bio.tools/polyphen) (23). were used. We also used VarSome Premium, which utilizes several in silico tools and provides a score for pathogenicity (https://doi.org/10.1093/bioinformatics/bty897) (24). Missense variants were finally evaluated according to the recommendations of the American College of Medical Genetics and Genomics (ACMG), and the Association for Molecular Pathology’s standards and guidelines (25).

### Variant validation

Sanger sequencing was performed to validate the novel variant detected and confirm segregation in the family. Oligonucleotide primers for PCR amplification of targeted variants were designed using Primer3 software (https://primer3.ut.ee/) and synthesized in-house at King Faisal Specialist Hospital and Research Centre (KFSHRC), Riyadh, Saudi Arabia. Sanger sequencing was performed as previously described (17). To ensure we have not missed variants in the most commonly mutated genes in FGD, *MC2R*, and *MRAP*, we also directly sequenced all exons and exon-intron boundaries of these two genes in the index case. No variant was detected in any amplicon of these two genes.

## Results

### Identification of a *TXNRD2* causal variant

**Figure 1:**
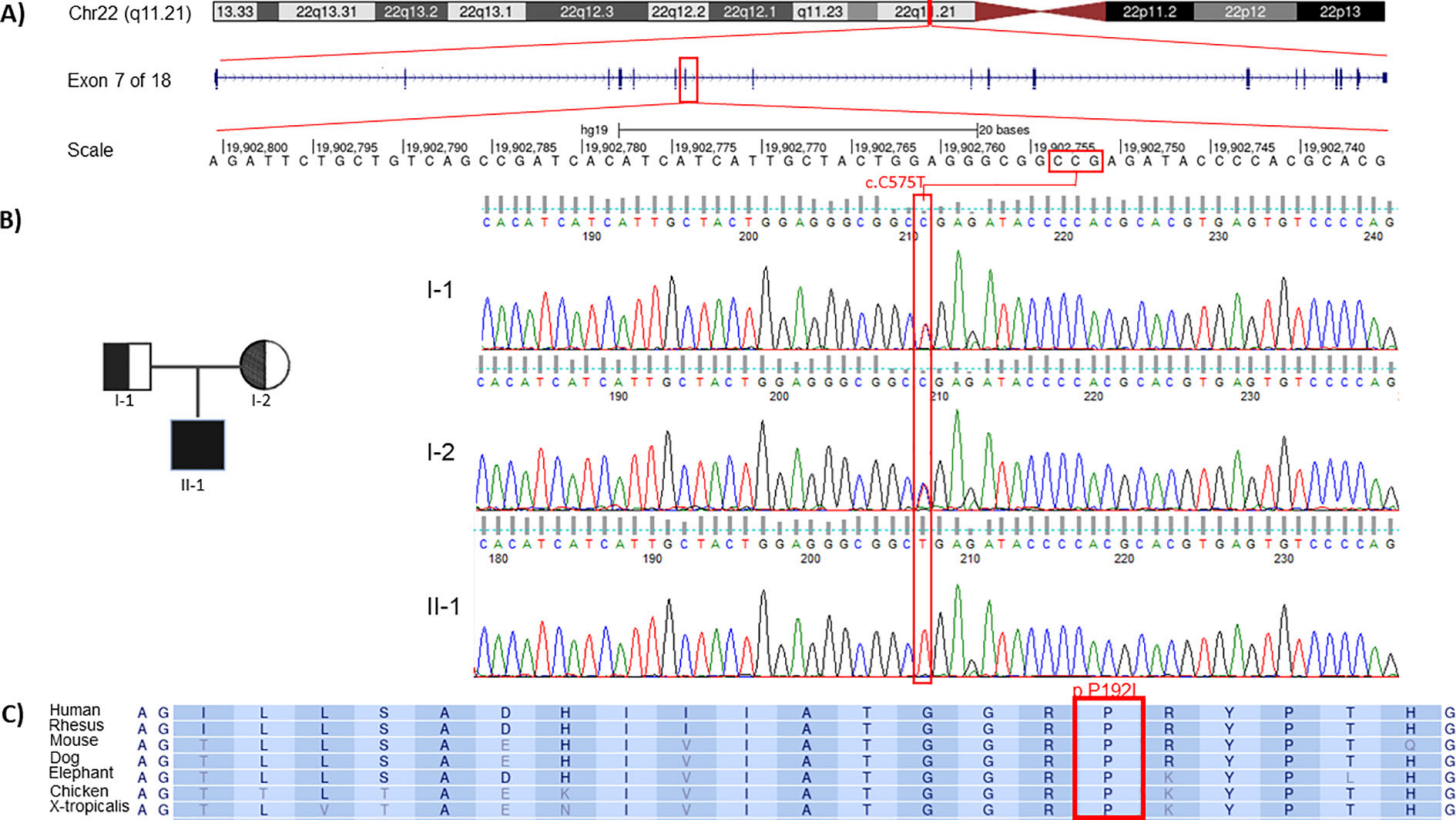
Molecular genetic analysis of the *TXNRD2* gene. **(A)** Schematic presentation of *TXNRD2* gene and localization of the identified variant (c.C575T) detected in an FGD patient. **(B)** Family pedigree generated using (https://www.progenygenetics.com), squares (males); circles (females); annotated symbols (affected individuals). Sanger sequencing chromatograms confirming the phenotype-causing missense c.575C>T variant in *TXNRD2* for index (II-1), index’s father (I-1), mother (I-2) all depicted by a red rectangle in the DNA sequence. **(C)** Alignment of the sequences of *H. sapiens* TXNRD2 with rhesus, mouse, dog, elephant, chicken and X-tropicalis.

To investigate the genetic cause of FGD, WGS was performed on the proband’s DNA. A novel missense variant in exon 7 of 18 of *TXNRD2* (NM_001282512) gene, c.575C>T (p.Pro192Leu), located at chr22:19,915,230, was identified (**Figure 1A**). The variant was homozygous in the affected individual, and segregation analysis revealed that the patient’s mother and father were heterozygous for the same *TXNRD2* variant, consistent with autosomal recessive inheritance (**Figure 1B**). This variant is extremely rare in population databases, with a reported allele frequency of ƒ = 0.00000479 in gnomAD and was not found in a local population database of 18,360 exomes. The affected Pro192 residue is highly conserved across multiple species, suggesting its functional importance (**Figure 1C**).

### In-silico classification of *TXNRD2* identified variant

*In-silico* analyzes were performed using commonly applied computational tools in accordance with ACMG recommendations. All prediction tools consistently showed that the c.575C>T (p.Pro192Leu) variant is deleterious. Of 32 *in silico* tools included in VarSome Premium software, 25 labelled this variant as pathogenic, 6 as variant of unknown significance (VUS) and only 2 as benign. The Meta scores were 12 pathogenic and 2 VUS. The affected highly conserved proline residue plays a critical structural role in protein folding due to its rigid cyclic structure (26), substitution with leucine is predicted to alter local protein conformation, potentially affecting protein stability or enzymatic activity. Based on ACMG/AMP guidelines, this variant was classified as VUS due to the absence of prior functional evidence and lack of previously established disease association. However, the *in silico* analysis and the impact on protein/enzymatic function are highly suggestive of its importance and the pathogenic effect of its change in this patient.

## Discussion

In this report, we describe a patient with FGD who has a novel genetic variant in the rarely mutated FGD-disease-causing gene, *TXNRD2*. The identified variant, located in exon 7 (c.575C>T; p.P192L), was detected in the homozygous state in the proband and in a heterozygous state in his consanguineous parents. This variant is a novel missense mutation that has not been previously reported in the literature, absent in local databases, and extremely rare in general population databases.

According to ACMG criteria, the variant is currently classified as VUS due to the lack of functional evidence and the absence of prior disease association. The identified variant affects a highly conserved amino acid residue (Pro192) across species, supporting its functional importance. Most *in-silico* prediction tools consistently predict deleterious effects on protein structure and folding due to its rigid cyclic structure, highly suggesting a potential pathogenic role. While computational predictions alone are insufficient to establish pathogenicity, the concordance of these findings, together with the absence of pathogenic/likely pathogenic or VUS in other FGD-associated genes in the right clinical context further supports its pathogenicity in the current patient.

*TXNRD2* encodes Thioredoxin Reductase 2, a selenoprotein enzyme located in the mitochondria. It maintains the cellular redox balance by reducing thioredoxins, which are essential for scavenging reactive oxygen species (ROS) and protecting against oxidative stress. It uses NADPH and selenocysteine residues to function, playing roles in cell signaling and protecting tissues with high metabolic demands, such as the heart and adrenal cortex, where steroidogenesis generates significant oxidative stress (27, 28). Given that *TXNRD2* is essential for redox regulation and protection against oxidative stress, disruption of TXNRD2 protein structure may impair its mitochondrial redox homeostasis function, leading to adrenal cortical dysfunction and impaired cortisol production, providing a plausible molecular mechanism for FGD.

Evidence supporting the role of TXNRD2 in human disease initially emerged from animal models. In 2004, work in which *TXNRD2* gene was disrupted in transgenic mice showed a phenotype reminiscent of human dilated cardiomyopathy (29). Subsequent screening for *TXNRD2* variants in a cohort of 227 patients with dilated cardiomyopathy identified 3 patients carrying 2 pathogenic variants in heterozygous state, which were absent in 683 controls (11). Since then, about 25 *TXNRD2* variants have been reported, with about half pathogenic and the other half strongly associated with disease, either found in mono- or bi-allelic forms, suggesting variable inheritance patterns and phenotypic expression (18). Diseases associated with *TXNRD2* variants include primary open-angle glaucoma, developmental delay, dilated cardiomyopathy and FGD (5, 9–14).

To date, only a limited reports on the role of *TXNRD2* in FGD, reporting only four variants, the missense variant c.1081G>A (p.V361M) (10), the non-sense variant c.1341T>G (p.Y447*) (13, 14), the splicing variant (c.1348-1G>T) (12, 13), and the compound heterozygous variants (c.1391A > G; p.H464R and c.1141C > T; p.R381W) (15) (Summarized in **Table 1**). Most reported disease-associated variants are loss-of-function missense variants affecting conserved residues or functionally important domains. However, the number of reported cases remains limited, and genotype–phenotype correlations are still emerging. The first report was published in 2014 and described seven siblings affected with FGD with segregation of the truncating *TXNRD2* mutation (c.1341T>G (p.Y447*) in all of them. Functional study of this variant showed impaired redox homeostasis in a human adrenocortical cell line (13). This same variant was subsequently reported in another patient diagnosed with FGD, who also presented with macrocephaly and seizures due to a c.1341T>G variant (14). Interestingly, this variant has also been identified in a heterozygous form in a 14-year-old girl with dilated cardiomyopathy but no adrenal insufficiency (30, 31). The authors hypothesized that the absence of cardiomyopathy in the previously reported 7-member family and the absence of FGD in their patient might reflect clinical heterogeneity, presence of a modifier gene that may have influenced the expression of the disease differently between the two families or due to oligogenic inheritance (presence of more than one gene causing the condition) (30). More recent reports have further expanded the clinical and molecular spectrum of TXNRD2-related FGD. Two additional reports of FGD cases due to *TXNRD2* variants were published in 2024 (10, 12). Patjamontri S. et al. described a 10-year-old boy of a Pakistani first-degree couple who presented with an undescended right testis, micropenis, and isolated glucocorticoid deficiency (10). WES identified a pathogenic variant *in TXNRD2* (c.1081G>A; p.V361M). Functional studies confirmed its impact on the expression and redox function of TXNRD2 (10). Brachet C. reported a 17.5-year-old-boy of Moroccan descent who presented at age 21 months with a seizure. He was found to have a small penis, diplegia, and neurodevelopmental delay and abnormalities, optic atrophy and increased white matter signals, especially in the corpus callosum (12). WES identified a splice-site mutation (c.1348-1G>T) leading to frameshift and truncation. Functional characterization of this variant showed increased production and a lack of mitochondrial ROS detoxification, with loss of cortisol production in patient-derived fibroblasts deprogrammed and differentiated to adrenal like cells carrying his variant (12). A recently reported case from a Chinese family further supports the involvement of *TXNRD2* in FGD. In that study, a compound heterozygous missense variants (c.1391A > G; p.H464R and c.1141C > T; p.R381W) in *TXNRD2* were identified in A 7-year-old Chinese male, who presented with isolated glucocorticoid deficiency and abnormal adrenal function. Functional analyzes demonstrated reduced TXNRD2 protein levels in a heterologous expression system, suggesting that impaired mitochondrial redox regulation underlies the disease mechanism (15). Similar to our findings, these reported variants, including homozygous missense variants and compound heterozygous variants that affect conserved residues, have been shown to diminish TXNRD2 protein levels or stability and, in some cases, to reduce enzyme expression and/or function. Together, these observations reinforce the critical role of TXNRD2 in adrenal steroidogenesis and provide additional evidence that missense variants disrupting mitochondrial redox homeostasis can contribute to the pathogenesis of FGD. The variability in clinical presentation across reported cases, including the presence or absence of extra-adrenal features, suggests a complex genotype–phenotype relationship. This may be influenced by variant type, residual enzymatic activity, genetic modifiers, or oligogenic inheritance.

From a broader perspective, disruption of mitochondrial redox homeostasis may have systemic implications, particularly in tissues with high energy demands. Genetic variants affecting these pathways may impact all tissues rich in mitochondria, resulting in a broad spectrum of disease manifestations. Within mitochondria, the thioredoxin and glutathione antioxidant systems, including TXNRD2, GPX1 (glutathione peroxidase 1), and PRDX3 (peroxiredoxin 3), rely on a continuous supply of NADPH generated by the proton-pumping nicotinamide nucleotide transhydrogenase (NNT), an essential protein of the inner mitochondrial membrane. In contrast to the ultra-rare *TXNRD2* variants, biallelic *NNT* variants are relatively common among patients with rare FGD (30). Recent studies in NNT-deficient patients have revealed expected extra-adrenal phenotypes involving puberty, fertility, cardiac function and thyroid function. These observations suggest that defects in mitochondrial antioxidant pathways may result in a broader multisystem phenotype and support the need for ongoing clinical surveillance (30).

Taken together, our findings expand the mutational and clinical spectrum of *TXNRD2*-related FGD. Although functional validation is lacking, the combination of genetic, computational, and clinical evidence provides a compelling rationale for the potential pathogenicity of this variant and underscores the importance of considering *TXNRD2* in the evaluation of unexplained adrenal insufficiency.

## Conclusion

In summary, we identified a homozygous *TXNRD2* missense variant in an adult patient with FGD and seizure disorder with changes in basal ganglia at diagnosis that resolved on follow up. The variant reported expands the mutational spectrum of this gene. The identified p.Pro192Leu variant affects a conserved residue and is predicted by *in silico* tools to have deleterious effects on TXNRD2 function. This lends further support to the limited available literature on the role of this gene in FGD and expands the repertoire of genetic variants in this critical gene for mitochondrial function and redox metabolism. Long-term follow-up of patients with *TXNRD2* variants is recommended as defective oxidative stress response may affect other tissues over time.

## Data Availability

Additional data will be provided by the corresponding author upon request.
